# Only one in four lactating mothers met the minimum dietary diversity score in the pastoral community, Afar region, Ethiopia: a community-based cross-sectional study

**DOI:** 10.1017/jns.2021.28

**Published:** 2021-06-01

**Authors:** Getahun Fentaw Mulaw, Fentaw Wassie Feleke, Kusse Urmale Mare

**Affiliations:** 1 School of Public Health, Woldia University, Woldia, Amhara, Ethiopia; 2 Department of Nursing, Samara University, Samara, Afar, Ethiopia

**Keywords:** Dietary diversity, Ethiopia, Lactating women, Meal frequency, Pastoralist community, DDS, dietary diversity score, MDDS, minimum dietary diversity score, WMDDS, women's minimum dietary diversity score, MMFW, minimum meal frequency for women, NGOs, non-governmental organizations, WHO, World Health Organization

## Abstract

Maternal dietary feeding practice is one of the proxy indicators of maternal nutrient adequacy and it improves outcomes for both mothers and their offspring. The minimum maternal dietary diversity score of lactating women is defined as when the mother ate at least four and above food groups from the nine food groups 24 h preceding the survey regardless of the portion size. Therefore, the present study aimed to determine the minimum dietary diversity score (MDDS) and its predictors among lactating mothers in the Pastoralist community, Ethiopia. A community-based cross-sectional study design was employed on 360 lactating mothers using a multi-stage sampling technique from 5 January 2020 to 10 February 2020. Data were collected using questionnaires and anthropometry measurements. Data were entered using EPI-data 4.6.02 and exported into SPSS version 25. Statistical significance was declared at *P*-value <0⋅05 at multivariable logistic regression. Only one in four lactating mothers met the MDDS. The majority of them consumed cereals in the preceding 24 h of data collection. The most important predictors were maternal meal frequency (adjusted odds ratio (AOR) 6⋅26; 95 % confidence interval (CI) (3⋅51, 11⋅15)), antenatal care (ANC) follow-up one to three times and four and above times (AOR: 2⋅58; 95 % CI (1⋅24, 5⋅36), 4⋅77 (1⋅90, 11⋅95), respectively) and secondary paternal education (AOR 2⋅97; 95 % CI (1⋅44, 6⋅11)). The MDDS among lactating mothers was low. Paternal education, maternal meal frequency and ANC follow-up were the significant predictors. Therefore, to improve maternal dietary diversity score emphasis should be given to those predictors.

## Introduction

At reproductive age, women in low- and middle-income countries are vulnerable to malnutrition^(1)^. However, maternal under-nutrition is a severe public health problem globally accounting for 45 % of all maternal deaths^(2)^. Moreover, physiologically the nutritional demand increases during lactation. During this phase, the requirements for both energy and essential nutrients are higher^(3)^.

Dietary practice is a major challenge in developing countries that take the line-share for the cause of under-nutrition^(1,4)^. A lack of access to an adequate diversified diet is identified as one of the severe problems among poor populations^(5)^. Dietary diversity (DD) is considered as the proxy indicator for measuring dietary adequacy among individuals^(6)^. Maternal under-nutrition is the major challenge, and it is the global agenda as central to health and sustainable development^(1)^.

Ethiopia is one of the low-income countries with the highest levels of malnutrition among lactating women in sub-Saharan Africa^(7,8)^. Similarly, the limited studies conducted from Ethiopia such as Aksum^(9)^ and South Gondar^(10)^ showed that DD of lactating mothers was low. Additionally, there is no documentation on the predictors of DD among lactating mothers in the Afar pastoralist community, Ethiopia. The present study aimed to determine the magnitude of meeting minimum dietary diversity score (MDDS) and its predictors among lactating mothers in Abala district, Afar pastoralist community, Ethiopia. This will help in designing a proper intervention to improve maternal nutrition and easily access information for further research about lactating mothers.

## Methods

### Study design, setting and period

A community-based cross-sectional study was conducted in Abala district, Afar region, Ethiopia. The district is located about 942 km northeast of Addis Ababa and 491 km far from the regional capital city, Samara. According to the projection of the 2007 national Census, the district has a total population of 43 372 with an area of 1188⋅72 km^2^. From the Abala health bureau report, the district has one general hospital, four health centres and eight functional health posts. The study was conducted from 5 January 2020 to 10 February 2020.

### Inclusion and exclusion criteria

The source population was all lactating women in the Abala district who had a child aged less than 24 months. Mothers were excluded if they were seriously ill and have physical deformities which alter the procedures to take correct anthropometric measurements.

### Sample size determination and sampling procedure

The sample size was calculated using the open EPI-info version 7.1.1 with considering the assumptions of 17⋅2 % magnitude of MDDS from a study done in Ghana^(11)^, 5 % margin of error, 95 % confidence interval (CI), 10 % non-response allowance and 1⋅5 design effect. The calculated final sample size was 362 lactating women.

### Sampling procedure

The study participants were selected using a multi-stage sampling technique. The Abala district has a total of 14 kebeles, from which 5 kebeles were selected randomly. The sample size was proportionally allocated based on the total number of lactating women in each selected kebele (from the health extension worker's family folder). Finally, study participants were selected using a systematic sampling method. In households where there are more than one lactating women, a lottery method was used to select one participant.

### Data collection procedure and quality control measures

A semi-structured questionnaire was developed through a critical review of relevant literature. The questionnaire was consisting of socio-demographic characteristics, maternal healthcare practice, maternal dietary feeding practice, sanitation and hygiene-related factors, and anthropometric measurements. Six diploma-holder health professionals as data collectors and two master-holder public health professionals as supervisors were recruited. Data were collected by direct face-to-face interviewing with lactating mothers and measuring anthropometry.

The dietary diversity of lactating women was collected using the women's dietary diversity score (WDDS). It is calculated by a simple count and summing-up of the number of food groups that an individual respondent has consumed over the preceding 24-h recall period regardless of the portion size from the nine food groups. The calculated MDDS is taken as the consumption of at least four or more food groups. A minimum meal frequency is also calculated by counting the frequency of meals an individual took 24 h before the survey^(12)^. The maternal mid upper arm circumference (MUAC) was measured using UNICEF measuring tape to the nearest 0⋅1 cm.

### Data quality control measures

The research questionnaire was prepared in the English version and translated into the local language (Afar af). Pre-testing was done on 10 % of the sample size in the none-selected kebeles of the Abala district. Data collectors and supervisors were selected based on their fluency in the local language and they were trained on data collection techniques.

The anthropometric measurement (MUAC of the lactating mother) was performed according to the World Health Organization (WHO) standardised procedures. It was measured by placing WHO MUAC measuring tape on the upper-middle arm between the acromion and olecranon process of the non-dominant hand. Duplicate anthropometric measurements were done in case of deviations from standard procedures in measuring to minimise measurement errors. Continuous supervision and follow-up of the data collectors were made to review and check for completeness and consistency of the collected data on daily basis. The collected data were handled and stored carefully and appropriately.

### Data processing and analysis

The data were cleaned and entered into the latest Epi-data version 4.6.02, and transferred to statistical package for social sciences (SPSS) version 25 software for statistical analysis. The study results were presented as mean (sd) or numbers (%).

The statistical association was determined using bivariable and multivariable logistic regressions were used. Statistical significance was determined using the adjusted and unadjusted odds ratio with 95 % CI and *P*-value <0⋅05. Predictor variables that have an association with the outcome variable at bivariable analysis with a *P*-value of <0⋅25 were selected and included in the multivariable logistic regression model. Analysis was done using a backward logistic regression model and, variables with a *P*-value < 0⋅05 in multivariable analysis were declared as statistically and independently significant predictors of under-nutrition among lactating mothers.

The final model was tested using Hosmer and Lemeshow's *χ*^2^ test *P*-value, and it was *P* = 0⋅821, which showed that the model was the best fit. The percentage of the model that was accurately classified was 82 % and the extent of multi-colinearity was also assessed using standard error cut off two, and no variables were found.

### Ethical considerations

The present study was extracted and analysed from a study conducted according to the guidelines laid down in the Declaration of Helsinki, and all procedures involving human subjects/patients were approved by the ethical review committee of Samara University College of Health and Medical Sciences (Ref: ERC0053/2019). Informed verbal consent was obtained from all subjects (verbal consent was witnessed and formally recorded).

### Operational definitions

- Minimum maternal dietary diversity score of lactating women
  - Met: If the mother ate at least four and above food groups from the nine food groups 24 h preceding the survey.
  - Not met: If the mother ate less than four food groups from the nine food groups 24 h preceding the survey.
- Minimum meal frequency:
  - Met: If the mother consumes at least four times and above times per 24 h preceding the survey, regardless of portion size.
  - Not met: If the mother consumes less than four times per 24 h preceding the survey, regardless of portion size.

- Maternal under-nutrition
  - Yes: if MUAC < 23 cm and
  - No: if MUAC ≥ 23 cm
- Lactating mother: A mother who was breastfeeding her child at the time of the survey.

## Result

### Socio-demographic characteristics of study participants

A total of 360 lactating mothers were included in the present study with a 99⋅4 % response rate. The mean (±sd) age was 29⋅68 (±7) years. The majority 297 (82⋅5 %) and 311 (85 %) of them were rural residents and Afar in ethnicity, respectively. More than half, 222 (61⋅7 %) of mothers did not receive a formal education (Table 1).

**Table 1.** Socio-demographic characteristics among lactating mothers in Abala district, Afar region, Northeast Ethiopia, 2020 (*n* 360)

Variables	Category	MDDS		Total *n* (%)
		Not met *n* (%)	Met *n* (%)	
Maternal age	15–24	71 (19⋅0)	33 (9⋅2)	104 (28⋅9)
	25–34	119 (33⋅1)	39 (10⋅8)	158 (43⋅9)
	35–49	85 (23⋅6)	13 (3⋅6)	98 (27⋅2)
	Mean (±sd)	(29⋅68 ± 7)		
Residence	Rural	240 (66⋅7)	57 (15⋅8)	297 (82⋅5)
	Urban	35 (9⋅7)	28 (7⋅8)	63 (13⋅5)
Ethnicity	Afar	248 (67⋅8)	63 (17⋅2)	311 (85⋅0)
	Others*	30 (8⋅0)	25 (6⋅9)	55 (15⋅0)
Religion	Muslim	247 (68⋅6)	63 (17⋅5)	310 (86⋅1)
	Orthodox	28 (7⋅8)	22 (6⋅1)	50 (13⋅9)
Maternal educational status	No formal education	187 (51⋅9)	35 (9⋅7)	222 (61⋅7)
	Primary	76 (21⋅1)	26 (7⋅2)	102 (28⋅3)
	Secondary and above	12 (3⋅3)	24 (6⋅7)	36 (10⋅0)
Maternal occupation	Pastoralist	147 (40⋅8)	21 (5⋅8)	168 (46⋅7)
	Housewife	88 (24⋅4)	32 (8⋅9)	120 (33⋅3)
	Merchant	30 (8⋅3)	15 (4⋅2)	45 (12⋅5)
	Employed***	10 (2⋅8)	17 (4⋅7)	27 (7⋅5)
Paternal educational status	No formal education	193 (53⋅6)	35 (9⋅7)	228 (63⋅3)
	Primary	47 (13⋅1)	13 (3⋅6)	60 (16⋅7)
	Secondary and above	35 (9⋅7)	37 (10⋅3)	72 (20⋅0)
Paternal occupation	Pastoralist	200 (55⋅6)	35 (9⋅7)	235 (65⋅3)
	Employed***	35 (9⋅7)	34 (9⋅4)	69 (19⋅2)
	Others ****	40 (11⋅1)	16 (4⋅4)	56 (15⋅5)
Family size	≤4	98 (26⋅8)	45 (12⋅3)	143 (39⋅1)
	>4	180 (49⋅2)	43 (11⋅7)	223 (60⋅9)
	Mean (±sd)	(5⋅64 ± 2⋅19)		
Decision maker of the household	Father	195 (53⋅3)	50 (13⋅7)	245 (68⋅1)
	Mother	41 (9⋅4)	12 (6⋅4)	53 (14⋅7)
	Jointly	39 (10⋅8)	23 (6⋅4)	62 (17⋅2)
Owning Television and/or Radio	Yes	34 (9⋅4)	25 (6⋅9)	59 (16⋅4)
	No	241 (66⋅9)	60 (16⋅7)	301 (83⋅6)
Farming land ownership	Yes	79 (21⋅9)	27 (7⋅5)	106 (29⋅4)
	No	196 (54⋅4)	58 (16⋅1)	254 (70⋅6)
Livestock ownership	Yes	240 (66⋅7)	59 (16⋅4)	299 (83⋅1)
	No	35 (9⋅7)	26 (7⋅2)	61 (16⋅9)

MDDS, minimum dietary diversity score.

*Tigray and Amhara; **Single, divorced and widowed; ***Governmental and NGOs; ****Daily labour.

### Lactating mothers health care and nutrition-related characteristics

More than one-third, 137 (38⋅1 %) of mothers were not attending antenatal care (ANC) follow-up. Of all study participants, institutional delivery for the index child was 97(26⋅9 %). The mean (±sd) score of maternal mid-upper arm circumference was 23⋅98 (±2⋅51) cm (Table 2).

**Table 2.** Health care-related characteristics among lactating mothers in Abala district, Afar region, Northeast Ethiopia, 2020 (*n* 360)

Variables	Category	MDDS		Total *n* (%)
		Not met *n* (%)	Met *n* (%)	
Antenatal care	No	124 (34⋅4)	13 (3⋅6)	137 (38⋅1)
	1–3	121 (33⋅6)	41 (11⋅4)	162 (45⋅0)
	≥4	30 (8⋅3)	31 (8⋅6)	61 (16⋅9)
Gravidity	Primigravida	39 (10⋅3)	24 (6⋅7)	63 (17⋅5)
	2–4	126 (35⋅0)	42 (11⋅7)	168 (46⋅7)
	≥5	110 (30⋅6)	19 (5⋅3)	129 (35⋅8)
Birth place	Home	216 (60⋅0)	47 (13⋅1)	263 (73⋅1)
	Health institution	59 (16⋅4)	38 (10⋅6)	97 (26⋅9)
Maternal MUAC	<23	108 (30⋅0)	11 (3⋅1)	119 (33⋅1)
	≥23	167 (46⋅4)	74 (20⋅6)	241 (66⋅9)
	Mean (±sd)	23⋅98 (±2⋅51)		

MDDS, minimum dietary diversity score.

#### Lactating mothers feeding practice

**Fig. 1.**
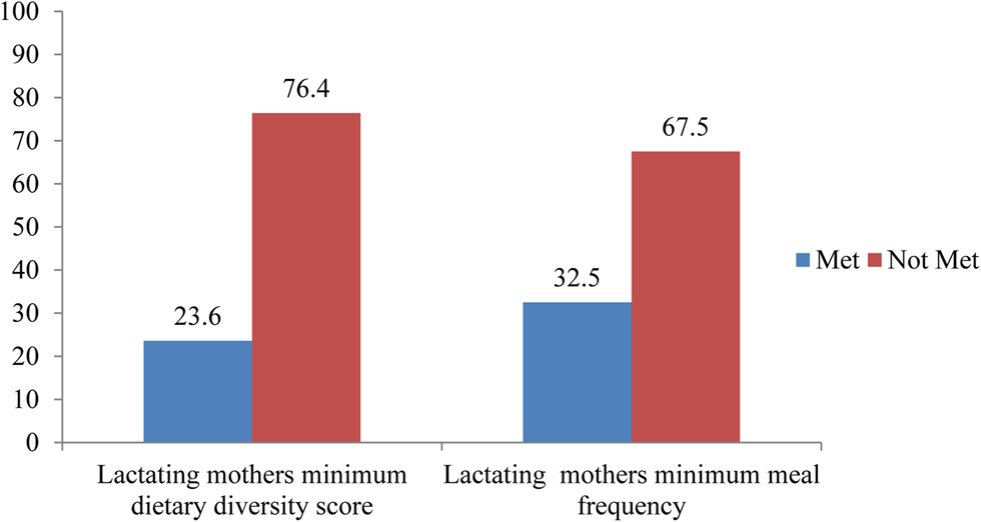
Percentage of women's minimum dietary diversity score (WMDDS) and minimum meal frequency for women (WMMF) among lactating mothers 24 h before the survey in Abala district, pastoralist community, Afar region, Northeast Ethiopia, 2020 (*n* 360).

**Fig. 2.**
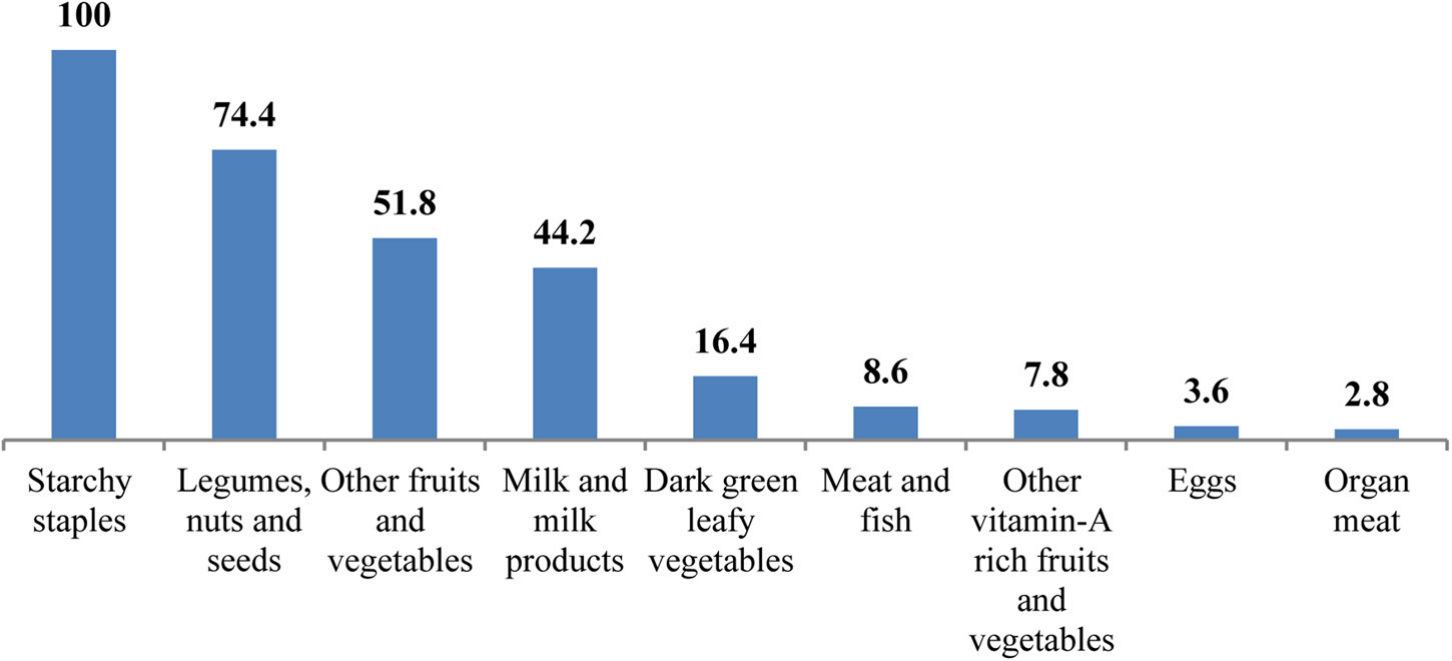
Percentage of food group consumption among lactating mothers 24 h before the survey in Abala district, pastoralist community, Afar region, Northeast Ethiopia, 2020 (*n* 360).

Less than two-third, 228(62⋅3 %) of study participants were getting nutrition-related counselling. There was food taboo during lactation in 7(1⋅9 %) of them. Two hundred and seventy-five (76⋅4 %) of study participants had a low dietary diversity score, while 84 (22⋅9 %) and 4 (1⋅1 %) of them had medium and high dietary diversity scores, respectively. The mean (±sd) score of maternal DDS and maternal meal frequency was (3⋅09 ± 0⋅875) and (3⋅33 ± 0⋅570), respectively. Only, 88 (24 %) of lactating mothers met the MDDS, while 117 (32⋅5 %) of them met the minimum meal frequency. In the last 24 h preceding the survey, all study participants consumed starchy staples, but only 16⋅4, 3⋅8 and 2⋅7 % of them had consumed dark green leafy vegetables, eggs and organ meat, respectively (Figs. 1 and 2).

#### Lactating mothers feeding practice one week before the survey

One week before the survey, all study participants consumed starchy staple foods and legumes. The least food groups consumed by study participants a week before were organ meat and other meat groups (Table 3).

**Table 3.** Proportion of lactating mothers who consume different food groups (at least one times) a week before the survey, in Abala district, pastoralist community of Afar region, Ethiopia, 2020 (*n* 360)

Food groups	MDDS		Total *n* (%)
	Met *n* (%)	Not met *n* (%)	
Starchy staples	275 (76⋅4)	85 (23⋅6)	360 (100⋅0)
Dark green leafy vegetables	108 (30⋅1)	72 (20⋅5)	180 (50⋅0)
Other vitamin A-rich fruits and vegetables	55 (15⋅3)	58 (16⋅1)	113 (31⋅4)
Other fruits and vegetables	250 (69⋅4)	84 (23⋅3)	334 (92⋅8)
Organ meat	14 (3⋅9)	14 (3⋅9)	28 (7⋅8)
Meat and fish	40 (11⋅1)	32 (8⋅9)	72 (20⋅0)
Eggs	42 (11⋅7)	33 (9⋅2)	75 (20⋅8)
Legumes, nuts and seeds	277 (76⋅4)	85 (23⋅6)	360 (100)
Milk and milk products	237 (65⋅8)	63 (17⋅5)	300 (83⋅3)

MDDS, minimum dietary diversity score.

#### Sanitation and hygiene-related characteristics of study participants

The majority 331 (91⋅9 %) of the study participants got water from protected sources, and more than half 203 (55⋅5 %) of them had no latrine. Nearly three-fourth, 271 (74⋅0 %) of study subjects dispose of solid waste at open fields (Table 4).

**Table 4.** Sanitation and hygiene-related characteristics of study participants in Abala district, the pastoralist community of Afar region, Ethiopia, 2020 (*n* 360)

Variables	Category	MDDS		Total *n* (%)
		Met *n* (%)	Not met *n* (%)	
Source of water	Protected	255 (70⋅8)	76 (21⋅1)	331 (91⋅9)
	Unprotected	20 (5⋅5)	9 (2⋅5)	29 (8⋅1)
Latrine ownership	Yes	110 (30⋅1)	53 (14⋅5)	158 (44⋅5)
	No	168 (45⋅9)	35 (9⋅6)	203 (55⋅5)
Type of latrine (*n* 158)	Pit latrine	95 (60⋅1)	38 (24⋅1)	133 (84⋅2)
	VIPL	12 (7⋅6)	13 (8⋅2)	25 (15⋅8)
Latrine functionality (*n* 158)	Yes	99 (62⋅7)	51 (32⋅3)	150 (94⋅9)
	No	8 (5⋅1)	0 (0)	8 (5⋅1)
Solid waste disposal site	Open-field (Inside the compound)	21 (5⋅8)	1 (0⋅3)	22 (6⋅1)
	Open-field (Outside the compound)	213 (58⋅2)	58 (15⋅8)	271 (74⋅0)
	Private pit	44 (12⋅0)	29 (8⋅0)	73 (20⋅0)
Soap usage for hand washing	Yes	43 (11⋅9)	25 (6⋅9)	68 (18⋅9)
	No	232 (64⋅4)	60 (16⋅7)	292 (81⋅1)

MDDS, minimum dietary diversity score; VIPL, ventilated improved pit latrine.

### Factors associated with MDDS

In the bivirate analysis variable like maternal residence, maternal age maternal educational status, maternal occupation, paternal occupation, family size, ANC follow-up, TV/radio ownership, maternal meal frequency, livestock ownership, gravidity, paternal education and birth place had significant association at *P*-value<0⋅25 with dietary diversity of lactating mothers (Supplementary Table S1 of Supplementary material),and entered into the multivariable logistic regression model to control the effect of confounders. Finally, paternal education, ANC follow-up and maternal meal frequency were maintained their consistency (Table 5).

**Table 5.** Factors associated with maternal dietary diversity score at multivariable logistic regression among lactating mothers in Abala district, Afar region, Northeast Ethiopia, 2020 (*n* 360)

Variables/Category	MDDS		COR (95 % CI)	AOR (95 % CI)
	Met	Not met		
Paternal education				
Not attend	35	193	1	1
Primary	13	47	1⋅53 (0⋅75, 3⋅11)	0⋅87 (0⋅39, 1⋅91)
Secondary and above	37	35	5⋅83 (3⋅25, 10⋅47)	2⋅97 (1⋅44, 6⋅11)**
Antenatal care follow-up				
No	13	124	1	1
1–3 times	41	121	3⋅23 (1⋅65, 6⋅33)	2⋅58 (1⋅24, 5⋅36)***
≥4 times	31	30	9⋅86 (4⋅61, 21⋅09)	4⋅77 (1⋅90, 11⋅95)**
Minimum meal frequency				
Met	56	61	6⋅77 (3⋅98, 11⋅52)	6⋅26 (3⋅51, 11⋅15)*
Not met	29	214	1	1

MDDS, minimum dietary diversity score; AOR, adjusted odds ratio.

*P < 0⋅001, **P < 0⋅005, ***P < 0⋅05.

## Discussion

The present study was done to determine the level of dietary diversity and its predictors among lactating mothers in Abala district, Afar pastoralist community National Regional State, Ethiopia. In the present study, the overall minimum dietary diversity among lactating mothers was 23⋅6 % (95 % CI 19 %, 28 %). The present study finding is similar to the study revealed in Oromia 23 %^(13)^.

The MDDS in the present study is lower than as compared with studies done in other parts of Ethiopia such as Angecha district 47⋅8 %^(14)^, Dedo and Seqa-Chekorsa district 32⋅8 %^(15)^, Ataye district 48⋅8 %^(16)^, Lay Gayt district 34⋅3 %^(17)^, Dessie town 45⋅5 %^(18)^, Aksum town 43⋅6 %^(9)^ and Debretabor 75 %^(19)^ showed that lactating mothers met the minimum diet diversity. This finding is also lower than findings from other low- to middle-income countries such as Nepal 53 %^(20)^ and Malawi 28⋅1 %^(21)^. In a case-control study in Dhaka, Bangladesh 42 %^(22)^ also has higher findings than the present study. But this finding is higher than from studies done in Leseto14⋅4 %^(23)^ and Ghana 17⋅2 %^(11)^. This difference might be due to sampling size, study design, setting, climate, tradition, poverty status and nutrition intervention.

According to the findings of the present study, the majority of lactating mothers consumed cereals in the preceding 24 h of data collection. It also showed that large proportions of the lactating mothers did not get source foods (egg, milk and milk products, organ meat, meat and fish). Additionally, a little proportion of lactating mothers consumed dark green leafy vegetables and other vitamin A-rich foods. This kind of feeding practice will expose mothers to different forms of malnutrition as evidenced from different studies^(14,21,22,24)^.

According to the present study, ANC follow-up, meal frequency and paternal education were the predictors for women's minimum dietary diversity score (WMDDS). The odds of meeting the minimum dietary diversity among women who attended ANC for one to three times and four and above times were 2⋅6 and 4⋅8 than those did not attend ANC follow-up services, respectively. The present study is consistent with the findings revealed from Nepal^(20)^. The possible explanation might be that participants who had attended ANC follow-up have nutritional counselling to utilise the diversified diet. It might be also due to higher education, more income and better diet on those who attended ANC follow-up^(21,25)^.

In the present study, mothers who met their minimum meal frequency were six times more likely to meet their MDDS than their counterparts. This is consistent with studies revealed in Ethiopia such as Lay Gayt district^(17)^ and Debretabor^(19)^. The possible reason might be a mother will met the minimum meal frequency if they are from households with food security. As a result, households secured with food might have met the minimum dietary diversity^(16)^.

Lactating mothers having paternal with the level of secondary educational status are more than two times more likely to met their dietary diversity than whose paternals’ do not have a secondary level of education. As the paternal educational status increases their nutritional knowledge might improve^(26)^, which can influence maternal feeding knowledge, attitude and practice^(16,27)^. Even though in the present study maternal education failed at the multivariable logistic regression model to be an important predictor, it has contributed to meeting minimum maternal diet diversity studies revealed from Ethiopian districts^(14,16)^.

### Limitations of the study

The present study is a cross-sectional study that cannot assess the cause–effect relationship. The seasonal variation in food consumption might exist so that results regarding dietary information are only limited to the specific season of the year in which the study was conducted. Beyond this, the study did not address the market access and barriers.

## Conclusion

The present study showed that more than three-fourths of the lactating mothers did not met their MDDS. Paternal education, ANC follow-up and maternal meal frequency were statistically independent predictors of dietary diversity. Therefore, interventions aimed at improving maternal dietary diversity should address those factors. Finally, the scientific community should study with a prospective cohort study design to address seasonal variability during the preharvest and postharvest seasons since dietary diversity is multifactorial to identify other independent predictors.

## Supplementary material

For supplementary material accompanying this paper visit http://dx.doi.org/10.1017/jns.2021.28. click here to view supplementary material

## References

[1] Black RE, Victora CG, Walker SP, (2013) Maternal and child undernutrition and overweight in low-income and middle-income countries. Lancet 382, 427–451. 23746772 10.1016/S0140-6736(13)60937-X

[2] Rush D (2000) Nutrition and maternal mortality in the developing world. Am J Clin Nutr 72, 212S–240S. 10871588 10.1093/ajcn/72.1.212S

[3] Meyers LD, Hellwig JP & Otten JJ (2006) Dietary Reference Intakes: The Essential Guide to Nutrient Requirements. Washington, DC: National Academies Press.

[4] World Health Organization (2010) *Indicators for Assessing Infant and Young Child Feeding Practices: Part 2: Measurement*. Available from: https://www.who.int/nutrition/publications/infantfeeding/9789241599290/en/

[5] Khan YM & Khan A (2012) A study on factors influencing the nutritional status of lactating women in Jammu, Kashmir and Ladakh regions. IJART 1, 65–74.

[6] Ruel MT (2003) Operationalizing dietary diversity: a review of measurement issues and research priorities. J Nutr 133, 3911S–3926S. 14672290 10.1093/jn/133.11.3911S

[7] Hazarika J, Saikia I & Hazarika PJ (2012) Risk factors of undernutrition among women in the reproductive age group of India: evidence from NFHS-3. Am Eur J Sci Res 7, 05–11.

[8] Hundera TD, Gemede HF, Wirtu D, (2015) Nutritional status and associated factors among lactating mothers in Nekemte Referral Hospital and Health Centers, Ethiopia. Int J Nutr Food Sci 4, 216–222.

[9] Weldehaweria NB, Misgina KH, Weldu MG, (2016) Dietary diversity and related factors among lactating women visiting public health facilities in Aksum town, Tigray, Northern Ethiopia. BMC Nutr 2, 38.

[10] Girma N & Degnet T (2015) Dietary diversity and associated factors among rural households in South Gondar Zone, Northwest Ethiopia. Feed the Future 5.

[11] Zakaria H & Laribick DB (2014) Socio-economic determinants of dietary diversity among women of child bearing ages in Northern Ghana. Food Science and Quality Management 34.

[12] Kennedy G, Ballard T & Dop MC (2011) Guidelines for Measuring Household and Individual Dietary Diversity. Rome, Italy: Food and Agriculture Organization of the United Nations.

[13] Bedada Damtie S, Benti Tefera T & Tegegne Haile M (2020) Dietary diversity practice and associated factors among children aged 6–23 months in Robe town, Bale zone, Ethiopia. J Nutr Metab 2020. 10.1155/2020/9190458 PMC7350076 32685209

[14] Boke MM & Geremew AB (2018) Low dietary diversity and associated factors among lactating mothers in Angecha districts, Southern Ethiopia: community based cross-sectional study. BMC Research Notes 11, 892. 30547839 10.1186/s13104-018-4001-6 PMC6295037

[15] Alemayehu M, Argaw A & Mariam AG (2015) Factors associated with malnutrition among lactating women in subsistence farming households from Dedo and Seqa-Chekorsa districts, Jimma zone, 2014. Develop Country Stud 5, 117–118.

[16] Getacher L, Egata G, Alemayehu T, (2020) Minimum dietary diversity and associated factors among lactating mothers in Ataye district, North Shoa zone, Central Ethiopia: a community-based cross-sectional study. medRxiv 2020. 10.1155/2020/1823697 PMC7817227 33520304

[17] Fentahun N & Alemu E (2020) Nearly one in three lactating mothers is suffering from inadequate dietary diversity in Amhara region, Northwest Ethiopia. J Nutr Metab 2020. 10.1155/2020/7429034 PMC7530474 33029395

[18] Seid A (2020) Dietary diversity, nutritional status and associated factors among lactating mothers visiting governmental health facilities of Dessie town, Amhara region, North Central Ethiopia. Doctoral Dissertation. 10.1371/journal.pone.0263957 PMC8853554 35176095

[19] Engidaw MT, Gebremariam AD, Tiruneh SA, (2019) Dietary diversity and associated factors among lactating mothers in Debre Tabor General Hospital, Northcentral Ethiopia. Int J 5, 17.

[20] Singh DR, Ghimire S, Upadhayay SR, (2020) Food insecurity and dietary diversity among lactating mothers in the urban municipality in the mountains of Nepal. PLoS ONE 15, e0227873. 31935272 10.1371/journal.pone.0227873 PMC6959598

[21] Kang Y, Hurley KM, Ruel-Bergeron J, (2019) Household food insecurity is associated with low dietary diversity among pregnant and lactating women in rural Malawi. Public Health Nutr 22, 697–705. 30378520 10.1017/S1368980018002719 PMC10260502

[22] Hasan M, Islam MM, Mubarak E, (2019) Mother's dietary diversity and association with stunting among children <2 years old in a low socio-economic environment: a case-control study in an urban care setting in Dhaka, Bangladesh. Matern Child Nutr 15, e12665. 30216672 10.1111/mcn.12665 PMC7199067

[23] Bonis-Profumo G, Stacey N & Brimblecombe J (2020) Maternal diets matter for children's dietary quality: seasonal dietary diversity and animal-source foods consumption in rural Timor-Leste. Matern Child Nutr 17, e13071. 32761775 10.1111/mcn.13071 PMC7729527

[24] Henjum S, Torheim LE, Thorne-Lyman AL, (2015) Low dietary diversity and micronutrient adequacy among lactating women in a peri-urban area of Nepal. Public Health Nutr 18, 3201–3210. 25824344 10.1017/S1368980015000671 PMC10271535

[25] Hundera TD, Gemede HF & Wirtu D (2015) Nutritional knowledge and determinant factors among lactating mothers in Nekemte referral hospital and health centers, East Wollega, Ethiopia. Food Science and Quality Management 38.

[26] Ambikapathi R, Passarelli S, Madzorera I, (2020) Men's nutrition knowledge is important for women's and children's nutrition in Ethiopia. Matern Child Nutr 17, e13062. 32755057 10.1111/mcn.13062 PMC7729551

[27] Agize A, Jara D & Dejenu G (2017) Level of knowledge and practice of mothers on minimum dietary diversity practices and associated factors for 6–23-month-old children in Adea Woreda, Oromia, Ethiopia. BioMed Res Int 2017. 10.1155/2017/7204562 PMC5405353 28497063

