# Supplementary material for: Only one in four lactating mothers met the minimum dietary diversity score in the pastoral community, Afar region, Ethiopia: a community-based cross-sectional study
Source: J Nutr Sci. 2021 Jun 1;10:e41. doi: 10.1017/jns.2021.28 (PMC8190715; doi:10.1017/jns.2021.28)
Supplement: Supplementary file 1 [file S2048679021000288sup001.docx]

Table S1: Factors associated with minimum dietary diversity score at bivariable logistic regression (at p-value < 0.25) among lactating mothers in Abala district, Afar region, North east Ethiopia, 2020 (n=360).

| **Variables / Category** | **MDDS** | | **COR(95% CI)** | | **P-value** |
| --- | --- | --- | --- | --- | --- |
|  | **Met** | **Not met** |  |  |  |
| **Maternal residence** | | | | |  |
| Rural | 57 | 240 | 1 | |  |
| Urban | 28 | 335 | 3.37(1.90 - 5.99) | | 0.000 |
| **Maternal age** | | | | | |
| 15-24 | 33 | 71 | 3.04(1.49-6.21) | 0.002 | |
| 25-34 | 39 | 119 | 2.14(1.08 – 4.26) | 0.03 | |
| 35-49 | 13 | 85 | 1 |  | |
| **Maternal education** | | | | | |
| Not educated | 35 | 187 | 1 |  | |
| Primary | 26 | 76 | 1.83(1.013 - 3.24) | 0.039 | |
| Secondary and above | 24 | 12 | 10.89(4.8 - 23.34) | 0.000 | |
| **Maternal occupation** | | | | | |
| Employed | 17 | 10 | 1 |  | |
| Housewife | 21 | 147 | 0.08(0.03 - 0.21) | 0.000 | |
| Merchant | 32 | 88 | 0.21(0.09 - 0.52) | 0.001 | |
| Pastoralist | 15 | 30 | 0.29(0.11 - 0.80) | 0.016 | |
| **Paternal education** | | | | | |
| Not attend | 35 | 193 | 1 |  | |
| Primary | 13 | 47 | 1.53(0.75 - 3.11) | 0.245 | |
| Secondary and above | 37 | 35 | 5.83(3.25 - 10.47) | 0.000 | |
| **Paternal occupation** | | | | | |
| Employed | 34 | 35 | 1 |  | |
| Pastoralist | 35 | 200 | 0.18(0.10 - 0.33) | 0.000 | |
| Others | 16 | 40 | 0.41(0.20 - 0.87) | 0.020 | |
| **Family size** | | | | | |
| <4 | 45 | 95 | 1.94(1.19 - 3.17) | 0.008 | |
| >4 | 42 | 180 | 1 |  | |
| **House hold decision** | | | | | |
| Husband | 50 | 195 | 1 |  | |
| Wife | 12 | 41 | 1.14(0.56 – 2.33) | 0.717 | |
| Together | 23 | 39 | 2.30(1.26 – 4.20) | 0.007 | |
| **Tv/radio ownership** | | | | | |
| Yes | 25 | 34 | 2.95(1.64 - 5.32) | 0.000 | |
| No | 60 | 241 | 1 |  | |
| **Livestock ownership** | | | | | |
| Yes | 59 | 240 | 3.02(1.69 – 5.41) | 0.000 | |
| No | 26 | 35 | 1 |  | |
| **ANC** | | | | | |
| No | 13 | 124 | 1 |  | |
| 1-3 times | 41 | 121 | 3.23(1.65 - 6.33) | 0.000 | |
| >4 times | 31 | 30 | 9.86(4.61 – 21.09) | 0.001 | |
| **Gravidity** | | | | | |
| Primigravida | 24 | 39 | 3.56(1.76 - 7.20) | 0.000 | |
| 2-4 | 42 | 126 | 1.93(1.06 - 3.51) | 0.032 | |
| > 5 | 19 | 110 | 1 |  | |
| **Minimum meal frequency** | | | | | |
| Met | 56 | 61 | 6.77 (3.98 - 11.52) | 0.000 | |
| Not met | 29 | 214 | 1 |  | |
| **Birth place** | | | | | |
| Home | 38 | 59 | 1 |  | |
| Health institution | 47 | 216 | 2.96(1.77 - 4.96) | 0.000 | |
